# Mastering your fellowship

**DOI:** 10.4102/safp.v62i1.5099

**Published:** 2020-02-20

**Authors:** Klaus B. von Pressentin, Mergan Naidoo, Wilhelm J. Steinberg, Lushiku Nkombua, Tasleem Ras

**Affiliations:** 1 Division of Family Medicine and Primary Care, Stellenbosch University, Cape Town, South Africa; 2 Department of Family Medicine, University of KwaZulu-Natal, Durban, South Africa; 3 Department of Family Medicine, University of the Free State, Bloemfontein, South Africa; 4 Department of Family Medicine, University of Pretoria, Pretoria, South Africa; 5 Division of Family Medicine, University of Cape Town, Cape Town, South Africa

**Keywords:** FCFP (SA), examination, assessment, family medicine, registrars, multiple choice questions, objectively structured clinical examination, evidence based medicine

## Abstract

The series, ‘Mastering your Fellowship’, provides examples of the Different question formats encountered in the written and clinical examinations, that is, Part A of the Fellowship of the College of Family Physicians of South Africa (FCFP [SA]) examination. The series is aimed at helping family medicine registrars prepare for this examination.

## Introduction

This section in the *South African Family Practice* journal is aimed at helping registrars prepare for the Fellowship of the College of Family Physicians of South Africa (FCFP [SA]) Final Part A examination and will provide examples of the question formats encountered in the written examination: multiple-choice question (MCQ) in the form of single best answer (SBA – Type A) and/or extended matching question (EMQ – Type R); short answer question (SAQ), questions based on the critical reading of a journal (evidence-based medicine) and an example of an objectively structured clinical examination (OSCE) question. Each of these question types are presented based on the College of Family Physicians blueprint and the key learning outcomes of the FCFP programme. The MCQs will be based on the 10 clinical domains of Family Medicine, the modified essay questions (MEQs) will be aligned with the five national unit standards and the critical reading section will include evidence-based medicine and primary care research methods.

This month’s edition is based on unit standard 1 (critically reviewing new evidence and applying the evidence in practice), unit standard 2 (evaluating and managing a patient according to the biopsychosocial approach) and unit standard 5 (conducting all aspects of healthcare in an ethical and professional manner). The theme for this edition is trauma and emergency. We suggest that you attempt answering the questions (by yourself or with peers or supervisors) before finding the model answers online: https://www.safpj.co.za/.

Please visit the Colleges of Medicine of South Africa website for guidelines on the Fellowship examination: https://www.cmsa.co.za/view_exam.aspx?QualificationID=9.

We are keen to hear about how this series is assisting registrars and their supervisors in preparing for the FCFP (SA) examination. Please email us your feedback and suggestions.

## Multiple-choice question: Single best answer

A 56-year-old male, a known hypertensive on enalapril 10 mg twice daily, hydrochlorothiazide 12.5 mg daily and amlodipine 10 mg daily, presents to the community health centre with headaches and dizziness. He claims compliance to treatment. His blood pressure (BP) = 180/110 (checked twice), heart rate = 90/min, respiratory rate = 18/min and temperature = 37 °C. He is alert with a Glasgow Coma Scale of 15/15, has no focal neurology or eye changes and examines normally except for a hyperdynamic apex beat in the fifth intercostal space, anterior axillary line. His urine dipsticks are normal. The most appropriate next step is to:

- Add carvedilol 12.5 mg twice daily and review in 48 hours.
- Add spironolactone 25 mg and review in 48 hours.
- Administer enalapril 10 mg and recheck BP in 1 hour.
- Administer diazepam 5 mg and refer to hospital.
- Start him on intravenous labetalol and refer to hospital.

*Answer*: (d)

This scenario is a common presentation in primary care and is often inappropriately managed. The standard treatment guidelines (STGs) for the Department of Health and the Hypertension (HT) Society Working Group provide a useful classification of patients presenting with Grade 3 HT, namely the following:

*Asymptomatic severe hypertension*: these patients are asymptomatic but have BP > 180/110 mmHg and may have evidence of progressive target-organ damage or complications. It is important to re-check after 1 h the BP after excluding obvious causes for the raised BP. If still elevated at the same level, one may commence oral therapy using a drug from a different class and follow up within a week.

*Hypertensive urgency*: this level of HT is symptomatic, usually with severe headache, shortness of breath and oedema. There are no immediate life-threatening complications. Ideally, all patients with this condition should be treated in a hospital. If the patient is not on treatment, commence two agents and aim to lower the diastolic BP to 100 mmHg slowly over 48–72 h. The agents recommended are calcium channel blockers, angiotensin-converting enzyme (ACE) inhibitors, beta-blockers (preferably not atenolol) and diuretics, respectively. The first option would be to administer diazepam 5 mg orally and ask the patient to rest for 1 h before checking the BP again.

*Hypertensive emergency*: this is severe, often acute elevation of BP associated with acute and ongoing damage to the kidneys, brain, heart, eyes (grade 4 retinopathy) or the vascular system. These patients need rapid (within minutes to hours) lowering of BP to safe levels (25% reduction) over a 24-h period if admission to the intensive care unit (ICU) is not possible. Hospitalisation is ideally in an ICU with experienced staff and regular monitoring. Alternatively, such patients may be managed in a high-care setting at a district hospital. Intravenous therapy individualised to the emergency is the current standard of care. Furosemide, labetalol, nitroprusside and nitro-glycerine are the preferred intravenous agents. Rapidly lowering the BP may result in stroke. Oral therapy is instituted once the BP is stable.

In the above scenario, the patient presents with a hypertensive urgency; therefore, the management would be to administer oral diazepam, recheck in 1 h and, if still elevated, commence a beta blocker such as carvedilol. This patient should be referred to hospital for assessment and ongoing monitoring as he would be classified as a case of resistant HT.

Further reading

- Hypertension guideline working group, Seedat YK, Rayner BL, Veriava Y. South African hypertension practice guideline 2014. Cardiovasc J Afr. 2014;25(6):288–294. https://doi.org/10.5830/CVJA-2014-062
- South African Department of Health. Hospital level standard treatment guidelines and essential medicines list [homepage on the Internet]. Pretoria: National Department of Health; 2015.
  EML App available from: https://play.google.com/store/apps/details?id=omp.guidance.phc&hl=af; https://itunes.apple.com/za/app/eml-clinical-guide/id990809414?mt=8
  Also available on EMguidance App: https://emguidance.com/.
- Kloeck WGJ, editor. A guide to the management of common medical emergencies in adults. 12th ed. Johannesburg: Academy of Advanced Life Support; 2017.

## Short answer question: The family physician’s role as care provider

You are the family physician on duty in the emergency department. A 28-year-old tree-feller is brought in after falling off a tree an hour ago. He fell from a height of 4 m. He is slightly drowsy and says he is very tired, but he is fully orientated. He claims to have numb legs. His BP is 96/45 mmHg, respiratory rate is 28/min and he is apyrexial. On secondary survey, you found that he has tenderness along his cervical spine. He has flaccid extremities and is unable to move them. There is no obvious bleeding, and his abdomen is soft and non-tender. There is no suggestion of long bone fractures.

(Total: 20 marks)

1. What additional information would be important to obtain from the emergency medical responders? (3)
2. What should be considered in the differential diagnosis and how would you initially manage these possibilities? (7)
3. Just as you think the patient has stabilised, he has an episode of apnoea and fits. In spite of attempts at resuscitation, he dies within 1 h of admission. You have recently been reminded of the severe shortage of organs in your province. What would be the necessary requirements (including legal) to harvest organs from this patient? (5)
4. His family members have just arrived at the hospital. How would you approach the family to secure the use of his organs? (3)
5. How would the ethical principle of autonomy be applied to this scenario? (2)

*Model answers*: This question was used in a previous FCFP (SA) examination.

1. **What additional information would be important to obtain from the emergency medical responders?** (1 mark each for any three of the sections well described; maximum 3 marks)
  - Reason of the fall: why/how/slip versus faint/fit.
  - Identified injuries: fall from height, for example, head injury, spinal injury, calcaneal injury, internal injuries, long bone fractures and soft tissue injuries.
  - What transpired after the fall? Was there loss of consciousness at the time, how long, any associated vomiting, amnesia, how long ago did this happen? What happened straight after the fall? Did he mobilise?
  - What emergency treatment was initiated? Was he mobilised or immobilised by others?
2. **What should be considered in the differential diagnosis and how would you initially manage these possibilities?** (maximum 7 marks)
  - Neurogenic shock from spinal injury – most likely. (1)
  - Hypovolemic shock from covert bleeding – a possibility. (1)
  - Manage above conditions by giving fluids carefully. Give bolus of 500 mL crystalloid and observe response. (1)
  - Immobilise spine – cervical collar and spinal board. (1)
  - Assess airway/breathing – monitor saturation, be ready for intubation and ventilation. (1)
  - Imaging – spinal X-rays in emergency centre (EC) (and possibly other trauma-related X-rays) and ultrasound (focussed assessment with sonography in trauma); also consider the need for computed tomography imaging. (1)
  - Constantly monitor BP/pulse/abdomen/haemoglobin/haemoglucose test. (1)
3. **What would be the necessary requirements (including legal) to harvest organs from this patient?** (maximum 5 marks)
  - There needs to be consent.
  - Preferably prior consent (‘living will’ or organ donation card).
  - If not available, the nearest relatives may consent as well.
  - Patient must be certified dead by two medical practitioners, one registered for at least 5 years and not part of the transplant team.
  - The organs need to be removed by a medical practitioner.
  - Must have an accredited donor institution in mind and may only be given to South African citizen/permanent resident.
  - No money may be paid for the receipt of the organ(s).
4. **His family members have just arrived at the hospital. How would you approach the family to secure the use of his organs?** (1 mark for each point, maximum 5 marks)
  - Find a quiet and private space to inform the family/relatives of the death. (1)
  - Give the family some space/time to consider. (1)
  - Ask about prior consent. (1)
  - Ask about organ donation – provide information, answer questions and refer to a counsellor if available. (1)
  - Follow steps of breaking bad news, with specific reference to issue of organ donation. (1)
5. **How would the ethical principle of autonomy be applied to this scenario?** (1 mark awarded for any three points raised from the possibilities below)
  - Autonomy – respect the prior autonomy of the deceased. Many may never have registered for organ donation but may recognise the ‘moral obligation of beneficence’, or to assist another when the harm to oneself is minimal or absent, and the value to others is great. However, some may refuse organ donation based on religious or other grounds. Some countries use an opt-out organ donation system to overcome this. (1)
  - Consent – South African law requires voluntary informed consent from the donor or next of kin. However, when a patient dies, doctors may find it difficult to speak to the bereaved family about removing organs in such an emotionally charged atmosphere. This includes the dilemma of having to face something in an emergency, which the doctors themselves have not yet fully worked through/accepted. Even when approached, the family may object, having just suffered the loss of a loved one and not knowing the deceased’s wishes in the matter. The family may need some time to talk about the possibility of organ transplant, yet are expected to provide an answer immediately (within hours). (1)

Further reading

- Moodley K. Family medicine ethics. In: Mash B, editor. Handbook of family medicine. 4th ed. Cape Town: Oxford University Press, 2017; p. 406–429.
- Republic of South Africa. National Health Act, Act 61 of 2003; Section 8. [cited 2020 Jan 23]. Available from: https://www.gov.za/documents/national-health-act
- Republic of South Africa. Human Tissue Act; no 65 of 1983; amended section 106 of 1984. [cited 2020 Jan 23]. Available from: http://www.kznhealth.gov.za/humantissueact.pdf

## Critical appraisal of quantitative research

Read the accompanying article carefully and then answer the following questions (total 25 marks). As far as possible, use your own words. Do not copy out chunks from the article. Be guided by the allocation of marks with respect to the length of your responses.

Kabale BM, Nkombua L, Matthews P, Offiong BE. Healthcare professionals’ perceptions of alcohol-intoxicated trauma patients: Implications for healthcare delivery at South Rand Hospital Emergency Department. S Afr Fam Pract [serial online]. 2013 [cited 2020 Jan 28];55(4):398–402. Available from: https://www.ajol.info/index.php/safp/article/view/94001

1. What were the authors’ key points in their argument for the social value of the study? (2 marks)
2. Comment on the title of the study in relation to the actual aim and content of the research article. (2 marks)
3. Critically appraise how the researcher describes the study design. (2 marks)
4. The researcher interviews 15 people by means of four focus group interviews. Discuss the pros and cons of this group approach versus individual interviews. (3 marks)
5. Several strategies are used to improve the trustworthiness of qualitative research. Critically appraise the use of the following in this study: (12 marks)
  - Peer briefing
  - Triangulation
  - Member checking
  - Purposive sampling
  - Audit trail
  - Reflexivity
6. Critically appraise any four of the recommendations. (4 marks)

*Model answers*: This question was used in the first semester 2019 FCFP [SA] examination.

1. **What were the authors’ key points in their argument for the social value of the study?** (2 marks) (Any one of the suggested answers = 1 mark)
  - Alcohol intoxication is one of the leading causes of morbidity and mortality in South Africa. (1)
  - Alcohol intoxication has been shown to increase the incidence of trauma as seen in patients treated at the research’s health facility. (1)
  - Data on alcohol-intoxicated trauma patients and their impact on healthcare practitioners were very limited. (1)
2. **Comment on the title of the study in relation to the actual aim and content of the research article.** (2 marks)
  - The title is misleading. In the title, the authors refer to trauma patients (trauma is broad: motor vehicle accidents, sport injuries, falls, interpersonal violence and assaults, etc.); however; they confined the study to alcohol-intoxicated assaulted patients. (1)
  - The study aimed to increase the awareness of healthcare professionals (HCPs) about their attitudes towards intoxicated patients, whereas the title suggests that the aim of the study was to assess HCP perceptions of alcohol-intoxicated patients. The title also suggests that a focus of the study is on implications for healthcare delivery – which the paper does not address. (1)
3. **Critically appraise how the researcher describes the study design. (2 marks) (Any two of the four points)** Usually, the study design is stated at the beginning of the methods section. Although this is not done (1), the researcher alludes to a ‘qualitative design’ in the Introduction of the article (1). This is however inadequate to position oneself within qualitative research traditions (1). It would be akin to describing a randomised controlled trial as a study with a ‘quantitative design’. It is necessary to characterise the study as, for example, phenomenology, ethnography or grounded theory. In this instance, the study belongs to a phenomenological tradition – exploring the meaning of a phenomenon of interest. (1)
4. **The researcher interviews 15 people by means of four focus group interviews. Discuss the pros and cons of this group approach versus individual interviews.** (3 marks) (Any three of the five points)
  Focus group interviews are usually made up of 8–15 people and therefore quite small (1). One expects that focus groups were used as a more efficient way of interviewing multiple people, as it would have taken longer to interview 15 people individually (1). Focus groups are influenced by the social dynamics between the people in the groups. Particularly powerful or outspoken individuals may influence the contribution of others (1). Individual interviews remove the effect of these social dynamics and ensure that the individual’s experience is fully explored (1). The power dynamics were limited to some extent by separating doctors and nurses into different groups (1).
5. **Several strategies are used to improve the trustworthiness of qualitative research. Critically appraise the use of the following in this study: (12 marks) (1 mark per topic, 1 for addressing the topic accurately and 1 for making a valid reflection)**
  - Peer briefing: a peer-review process is mentioned, but what was done is unclear. This might imply that the data analysis process and interpretation of data were reviewed by a panel of peers, which might be the other researchers. This would be done so that peers could provide feedback on the validity of the analysis.
  - Triangulation: the researcher explains that there was a ‘mental triangulation of findings’, but it is unclear what is meant by this. If this was an internal mental process in the mind of the researcher, then it is not really a triangulation. Triangulation requires results from different data sources (there are nurses and doctors in this study), types of data (it is all the same) or methods of data collection (all collected in the same way) to be juxtaposed in order to enhance the understanding of the phenomenon.
  - Member checking: this is also mentioned, but it is not clear what was checked. Was this just checking whether the transcription was accurate or whether the interpretation of the data was valid? Also known as respondent validation, this would imply some kind of feedback to the participants. However, hsow this was done is not explained.
  - Purposive sampling: this implies that people are purposefully selected to ensure the phenomenon is fully explored. Various types of sampling are possible, such as extreme case, criterion, snowball or random. Several criteria are mentioned, and it appears that these were used to ensure a variety of people were interviewed. The sample contains more doctors than nurses, while the EC staff probably had more nurses. It is also not explained why 15 people were considered enough to explore the phenomenon. Usually, the researcher would explain how they ensured saturation of themes.
  - Audit trail: this refers to the extent to which an outsider can follow the trail from raw data to findings. It is clear that interviews were recorded, transcribed verbatim and checked for errors. The process after that is rather confusing. The findings present ‘themes’ while the analysis process starts with ‘themes’ and then proceeds to ‘codes’ and finally ‘categories’. It appears that each transcript was analysed separately by two people and then the interpretations compared. No particular method is referenced for the analysis. It would be difficult for someone to reconstruct what was actually done.
  - Reflexivity: The researchers inform us that they are staff members but do not give a full picture of their relationship with the interviewees. For example, what was their gender and professional role in the team? Position in the hierarchy and degree of power in relationships may influence how people respond. The researcher asserts that their preconceptions were explored, but how this was done is not explained in the methods.
6. **Critically appraise any four of the recommendations. (4 marks) (1 mark per recommendation with a valid reflection, out of seven possible options)**
  - The discussion suggests that dealing with intoxicated patients may increase emotional stress and burnout amongst HCPs and recommends debriefing, counselling and psychosocial support. Whilst this seems like a logical linkage, the study does not provide evidence for this and does not reference evidence that links stress from intoxicated patients with burnout. This recommendation is probably valid for those suffering from burnout but may not be for all HCPs working in ECs.
  - The discussion also suggests that providing more staff would decrease emotional stress and burnout. This may be true in terms of reducing workload, but the study does not look at the relationship between workload and burnout. Is there evidence that seeing fewer intoxicated patients would reduce emotional stress and reduce burnout? The recommendation may be valid, but again it is not directly supported by the findings or the evidence cited.
  - The study also suggests that intoxicated patients may be treated poorly and stigmatised. The researchers suggest that this behaviour may be minimised by debriefing, counselling and psychosocial support. Again, this is an assumption and no evidence is provided to support this hypothesis.
  - The discussion suggests that alcohol abuse needs to be addressed at multiple levels because of the financial burden it places on healthcare. It is certainly true that treating patients in the EC is not addressing the roots of the problem of alcohol use in the South African society. The HCPs’ experience is one of the symptoms of the alcohol problem in South Africa and intervention here will not have impact on the problem.
  - The discussion suggests that providing training on communication, cultural diversity, conflict resolution and stress management will be helpful. Again, this is probably true in general. No evidence was cited to back up the idea that this kind of training would improve quality of care in the EC for intoxicated patients.
  - The discussion suggests that protective clothing must be provided to reduce the risk of injury or infection. This is practical and flows directly from the findings. Is there a problem with a lack of such clothing and, if so, what exactly needs to be provided?
  - The study also suggests that security in the EC and management of the escorts is a problem. Practical recommendations are suggested for infrastructure and guidelines for security staff. This also flows directly from the findings.

(Total: 25 marks)

Further reading

- Mabuza LH, Govender I, Ogunbanjo GA, Mash B. African primary care research: Qualitative data analysis and writing results. Afr J Prim Health Care Fam Med. 2014;6(1):1–5. https://doi.org/10.4102/phcfm.v6i1.640
- Kuper A, Lingard L, Levinson W. Critically appraising qualitative research. BMJ. 2008;337:a1035. https://doi.org/10.1136/bmj.a1035
- CASP Checklists. Critical appraisal skills programme [homepage on the Internet]. c2018. [cited 2020 Jan 28]. Available from: https://casp-uk.net/casp-tools-checklists/
- The Center for Evidence-Based Management. Critical appraisal of a qualitative study. Resources and tools [homepage on the Internet]. c2018. [cited 2020 Jan 28]. Available from: https://www.cebma.org/resources-and-tools/

## Objectively structured clinical examination scenario

**Objective of station**

- This station tests the candidate’s ability to manage a patient with advanced chronic obstructive pulmonary disease (COPD) in the EC.

**Type of station**

- Integrated consultation – clinical management, complex consultation.

**Equipment list**

- Role-player – adult male in his 50s.

### Instructions for candidate

#### History and context

You are the family physician working in the EC at a district hospital. The following patient is well known to the facility with advanced COPD. The nurse has already given him his nebulisation and a dose of oral prednisone. Consult with this gentleman and develop a comprehensive plan for his ongoing management. You do not need to perform a physical examination. Physical examination findings and investigations will be provided to you on request.

### Instructions for the examiner

**Objectives:** This station tests the candidate’s ability to manage a patient with advanced COPD in the EC.

This is an integrated consultation station in which the candidate has 14 min.

Familiarise yourself with the assessor guidelines that detail the required responses expected from the candidate.

No marks are allocated. In the mark sheet, tick off one of the three responses for each of the competencies listed. Make sure you are clear on what the criteria are for judging a candidate’s competence in each area.

Provide the *examination findings and investigations* to the candidate when requested.

**FIGURE 1:**
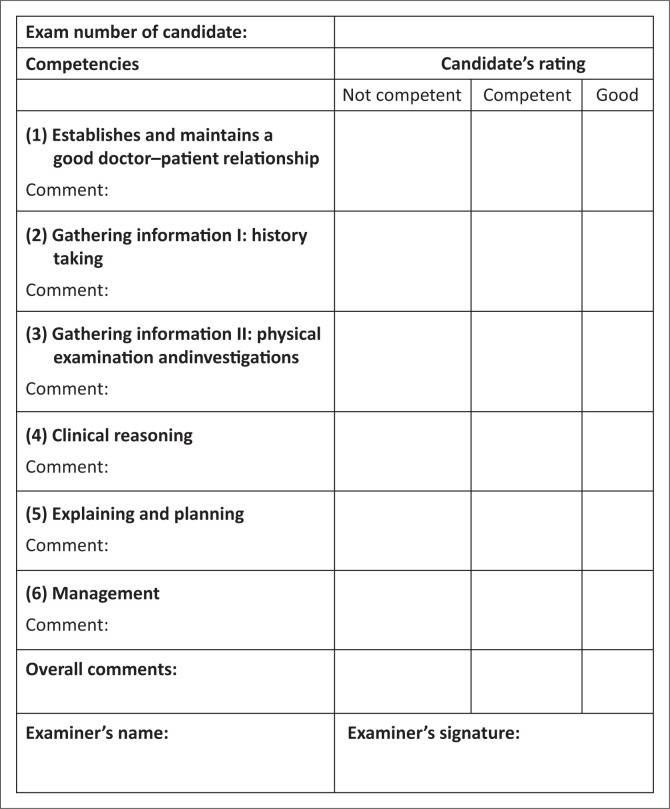
Marking template for consultation station.

This station is 15 min long. The candidate has 14 min, then you have 1 min between candidates to complete the mark sheet (Figure 1) and prepare the station.

Further reading

- South African Department of Health. Chapter 16.4. Hospital level standard treatment guidelines and essential medicines list. Pretoria: National Department of Health; 2015.
- NICE Guideline [NG115]. Chronic obstructive pulmonary disease in over 16s: Diagnosis and management [homepage on the Internet]. 2019 [cited 2020 Jan 28]. Available from: https://www.nice.org.uk/guidance/ng115
- Abdool-Gaffar MS, Ambaram A, Ainslie GM, et al. Guideline for the management of chronic obstructive pulmonary disease: 2011 update. S Afr Med J. 2011;101(1):63–73. https://doi.org/10.7196/SAMJ.4490
- Govender I, Okonto HI, Rangiah S, Nzaumvila D. Management of the patient with chronic obstructive airway disease (COPD) in a primary health care context. S Afr Fam Pract. 2019;61(5):10–14.
- Viviers PJ, Van Zyl-Smit RN. Chronic obstructive pulmonary disease-diagnosis and classification of severity. S Afr Med J. 2015;105(9):786–788. https://doi.org/10.7196/SAMJnew.8421

## Guidance for examiner

### Some general descriptors of competencies

*Working definition of competent performance*: the candidate *effectively completes the task* within the allotted time, in a manner that *maintains patient safety*, even though the execution may not be efficient and well structured.

### Establishes a good doctor–patient relationship

Shows genuine respect, compassion, sensitivity, rapport, empathy; establishes trust; and attends to patient’s comfort. Acknowledge patient’s discomfort and the anxiety related to ongoing physical symptoms.

A *competent candidate* acts within the ethical framework (respects autonomy, justice, non-maleficence and beneficence). In addition, the *good candidate* displays empathy and compassion.

### Gathering information: History

The *competent candidate* gathers enough information to identify current medical issues (*severe functional impairments; ongoing smoking pre-contemplative; repeated emergency centre consults with no comprehensive assessment*) and identifies any ongoing biopsychosocial risks. In addition, the *good candidate* explores the patient’s experience, fears (*fear of death*) and expectations, health-seeking behaviour (*misses outpatient department [OPD] and pharmacy dates*) and identifies opportunities for health promotion (*improved relationships, advanced care planning*).

### Gathering information: Examination and investigations

*The competent candidate* would elicit enough information to identify the key issues (*peripheral oedema; raised* jugular venous pressure [JVP]*; centrally cyanosed; hyperinflated;* chest X-ray; electrocardiogram *with right ventricular hypertrophy*) and identify any ongoing biopsychosocial issues. In addition, *the good candidate* demonstrates rational use of investigations (*blood gas, haemoglobin* and *spirometry*).

### Clinical judgement

The *competent candidate* uses available evidence to make the correct working diagnosis (*end-stage COPD with cor pulmonale*). The *good candidate* can make a comprehensive three-stage assessment (*end-stage COPD with cor pulmonale, inappropriate use of the emergency centre, fear of death, incomplete tasks related to family relationships*) or use a standardised model, for example, Stott’s model.

### Explaining and planning

The *competent candidate* clearly explains the working diagnosis (*no jargon, comprehensive, simple language*) and possible interventions. The *good candidate*, in addition, provides a platform for the patient to engage as an equal partner in sharing information and decision-making.

### Management

The *competent candidate* uses current evidence-based guidelines to develop a management plan. In addition, the *good candidate* develops a comprehensive plan within a palliative care framework, for example, using the bio-psycho-socio-spiritual approach.

## Additional guidance for examiner

### Acute management of a chronic obstructive pulmonary disease exacerbation

- Nebulisation – start with salbutamol, add ipratropium bromide.
- Oral prednisone if able to swallow (alternative intravenous hydrocortisone).
- Antibiotics not routinely indicated but if considered necessary (moderate to severe symptoms with > 2 or more of the following symptoms – increased dyspnoea, cough, sputum production – especially if purulent) start with amoxicillin. If there is evidence of recent antibiotic use (last 3 weeks), give co-amoxiclav or azithromycin (if severe penicillin allergy).

### Post-acute

- Behavioural:
  ■ Proper use of primary care facilities.
  ■ Smoking cessation – although debatable in a potentially stage 4 COPD patient.
  ■ Optimise chronic medication.
- Clinical:
  ■ Consider long-acting bronchodilator/inhaled corticosteroid combo.
  ■ Consider theophylline orally.
  ■ Consider formal spirometry for disease grading.
  ■ Involve multidisciplinary team (MDT).
- Manage *cor pulmonale* optimally:
  ■ Diuretic – furosemide.
  ■ Consider ACE-inhibitor.
  ■ Consider spironolactone.
  ■ Consider cardio-selective β-blocker.
- Adopt a palliative care approach:
  ■ Control breathlessness and anxiety: mist morphine and bensodiazepine (e.g. lorazepam).
  ■ Involve family and spiritual counsellor.
  ■ MDT approach.
  ■ Advanced care planning.

## Examination findings and investigations

Add the relevant details for the examiner – examiner *should not* show all the examination findings to the candidate but should respond to specific questions being asked.

General examination:

- BP 100/75
- Pulse 98/min – weak peripherally; regular
- Centrally cyanosed
- JVP raised: 4 cm
- Peripheral oedema: ankles.

Respiratory system:

- Hyper-resonance to percussion bilaterally
- Decreased air-entry bilaterally, with widespread expiratory wheezes.

Cardiovascular system:

- Soft heart sounds
- No other abnormal findings.

Abdomen:

- Slightly distended – no specific findings.

**FIGURE 2:**
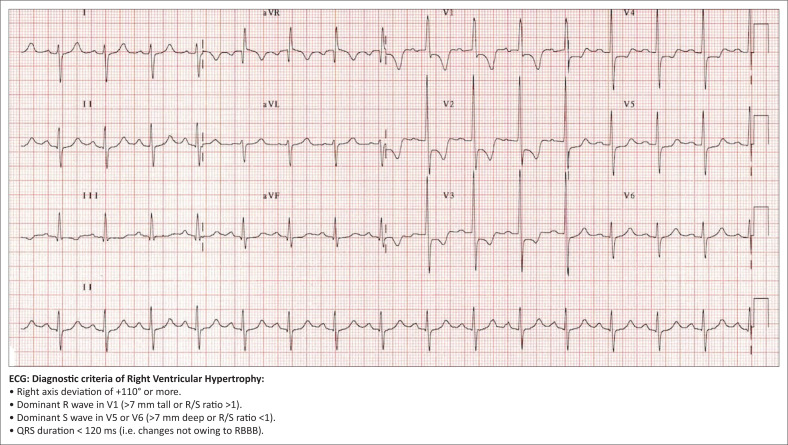
Electrocardiogram for objectively structured clinical examination station.

**FIGURE 3:**
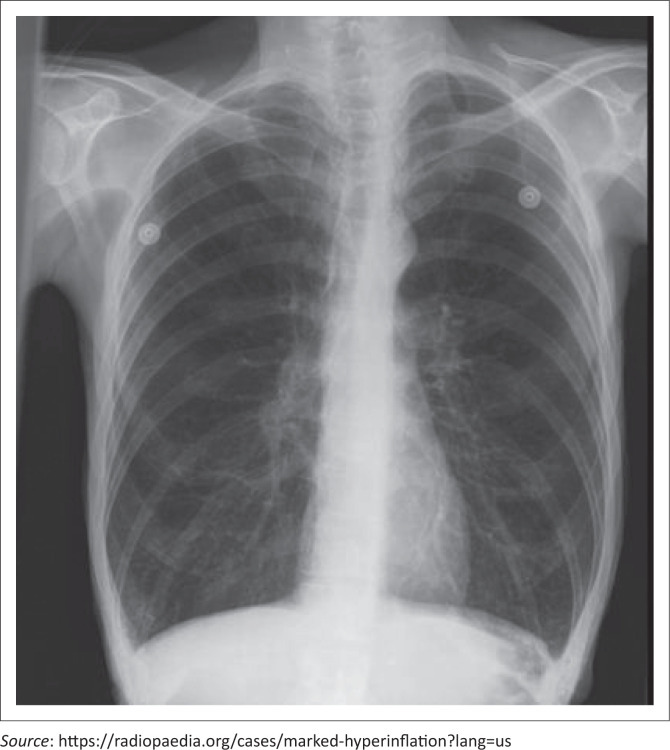
Chest X-ray for objectively structured clinical examination station (main finding: only hyperinflated).

Examiner *may show* the results of investigations/radiology to the candidate (see Figures 2 and 3).

## Role–play: Instructions for actor

**Appearance and behaviour**

Neat, well-groomed gentleman in his late 50s. Unable to speak continuously owing to shortness of breath: five to six words, then pauses to catch breath.

**Opening statement**

‘Doctor, I just need my pump and prednisone, then I will go home’.

### History

Open responses – Freely tell the doctor

- You have COPD for the last 5 years.
- Need to visit the EC 2–3 times/week when the pump does not work.

**Closed responses**

Only tell the doctor if asked:

- Get all your meds via EC visits – long time since been in OPD or clinic.
- Smoking – not willing to stop – it calms you down.
- Get very anxious and scared most of the time.
- No other medical problems. Not on any other medication.

Ideas, concerns and expectations:

- Fears that one day you will die from this problem.
- Worried what will happen to your family – understand that there is serious damage to your lungs.

Medical history:

- Besides this problem, nothing else.

Family and social history:

- Unable to work – on disability grant for last 2 years.
- Children all grown up.
- Wife died of cancer last year.

